# Inhibition of the activity of poly (ADP-ribose) polymerase reduces heart ischaemia/reperfusion injury *via* suppressing JNK-mediated AIF translocation

**DOI:** 10.1111/j.1582-4934.2008.00183.x

**Published:** 2008-08-11

**Authors:** Zhao-Feng Song, Xiao-Ping Ji, Xiao-Xing Li, Sheng-Jun Wang, Shu-Hua Wang, Yun-Zhang

**Affiliations:** a Department of Cardiology, Shandong University Qilu HospitalJinan, Shandong Province, China; b The Key Laboratory of Cardiovascular Remodeling and Function Research of Chinese Ministry of Education and Public Health, Shandong UniversityJinan, Shandong Province, China; c Department of Cardiology, Taian Central HospitalTaian, Shandong Province, China; d Department of Neurology, Shandong University Qilu HospitalJinan, Shandong Province, China

**Keywords:** PARP inhibition, heart ischaemia/reperfusion, apoptosis-inducing factor, c-Jun NH2-terminal kinase

## Abstract

Poly (ADP-ribose) polymerase (PARP) has been proposed to play an important role in the pathogenesis of heart ischaemia/reperfusion (I/R) injury. However, the mechanisms of PARP-mediated heart I/R injury *in vivo* are still not thoroughly understood. Therefore, in this study, we investigate the effect of PARP inhibition on heart I/R injury and try to elucidate the underlying mechanisms. Studies were performed with I/R rats' hearts *in vivo*. Ischaemia followed by reperfusion caused a significant increase in Poly (ADP-ribose) (PAR), c-Jun NH2-terminal kinase (JNK) and apoptosis-inducing factor (AIF) activity. Administration of 3,4-dihydro-5-[4-(1-piperidinyl)butoxy]-1(2H)-isoquinolinone (DPQ), an inhibitor of PARP, decreased myocardial infarction size from 61.11±7.46%[0] to 38.83±5.67% (P<0.05) and cells apoptosis from 35±5.3% to 20±4.1% (P<0.05) and simultaneously improved the cardiac function. Western blot analysis showed that administration of DPQ reduced the activation of JNK and attenuated mitochondrial-nuclear translocation of AIF. Additionally, administration of SP600125, an inhibitor of JNK, attenuated mitochondrial-nuclear translocation of AIF. The results of the present study demonstrated that the inhibition of PARP was able to reduce heart I/R injury *in vivo*. Our results also suggested that JNK may be downstream of PARP activation and be required for PARP-mediated AIF translocation. Inhibition of the activity of PARP may reduce heart I/R injury via suppressing AIF translocation mediated by JNK.

## Introduction

Poly (ADP-ribose) polymerase (PARP) is a chromatin-bound enzyme that plays a physiological role in the repair of strand breaks in DNA [1]. PARP locates in the nuclei of cells of various tissues, including the heart and skeletal muscle [2]. The breakage of DNA strands can activate PARP. The activation of PARP initiates an energy-consuming cycle by transferring ADP ribose units from NAD^+^ to nuclear proteins [3, 4]. As a result, the damaged DNA was repaired. However, in many pathophysiological processes, such as inflammation, circulatory collapse and heart ischaemia/reperfusion (I/R) injury, massive amounts of reactive oxygen species, including superoxide anions [5], hydrogen peroxide and hydroxyl radicals [6], are generated, which cause the breakage of DNA strands and subsequently hyper-active of PARP. This process leads to the depletion of the intracellular nicoti-namide adenine dinucleotide (NAD^+^) and ATP energetic pools which leading to cellular dysfunction and death.

Heart I/R may exacerbate myocardial injury by the release of massive of reactive oxygen and nitrogen species. These reactive oxygen and nitrogen species are able to lead to cell necrosis and apoptosis. PARP has been proposed to play a role in the pathogen-esis of heart I/R injury [7, 8]. Poly (ADP-ribose) (PAR) is the active morphous of PARP. Immunohistochemical detection of PAR formation demonstrated that PARP was activated in the reperfusion myocardium [9]. Myocardial reperfusion injuries *in vitro* or *in vivo* have been shown to ameliorate by pharmacological inhibitors of PARP [10, 11], which indicated that the PARP pathway is involved in the pathogenesis of heart I/R injury.

Apoptosis-inducing factor (AIF) is a novel apoptotic factor that induces chromatin condensation and large-scale DNA fragmentation. AIF is a caspase-independent mitochondrial death factor [12]. AIF is strictly confined to mitochondria in normal cells. The most conspicuous feature of AIF in apoptosis is its mitochondrial-nuclear translocation, accompanied by nuclear condensation and large-scale DNA degradation [13]. In oxidant-induced cell, AIF transposes to the nucleus and induces cell death. Studies demonstrated that heart I/R *in vivo* were associated with the nuclear translocation of AIF [12]. Studies also demonstrated that PARP regulated the translocation of AIF, which contributed to myocardial injury and cardiac dysfunction in heart I/R [14, 15].

Though many studies have reported that PARP hyper-activation prompted mitochondria dysfunction, which in turn released AIF and subsequently led to cell death [16], the mechanism by which PARP activation led to mitochondrial dysfunction and released of AIF was largely unknown [17, 18]. Recently, Xu et al. [19] found that c-Jun NH2-terminal kinase (JNK) participated in PARP-mediated mitochondria dysfunction and subsequently cell death in mouse embryonic fibroblast cells. However, the role of JNK in PARP mediates mitochondria dysfunction and the releases of AIF *in vivo* are still unknown. Therefore, the aim of the present study is to investigate whether JNK participates in the PARP mediates release of AIF in I/R hearts *in vivo*.

## Materials and methods

### Myocardial ischaemia and reperfusion

The investigation conformed to the local guidelines for the animal care and use committees. Female Wistar rats (250–350 g body weight; from Shandong University, China) were anaesthetized with sodium pentobarbi-tal (50 mg/kg i.p.). The trachea was cannulated with a PE-90 catheter and artificial respiration was provided by a respirator with FiO2 of 0.80, a frequency of 100 strokes/min. and a tidal volume of 0.8–1.2 ml to maintain normal PO_2_, PCO_2_, and pH. A left thoracotomy and pericardiotomy were performed. The left anterior descending branch (LAD) of the left coronary artery was occluded by ligation with a 4–0 silk suture. After 60 min. of ischaemia, the ligation was loosened and reperfusion occurred. The sham control animals were subjected to the entire surgical procedure and silk suture was passed beneath the coronary artery but the LAD coronary artery was not ligated. Rats were killed at 1 hr, 3 hrs, 6 hrs, 12 hrs and 24 hrs of reperfusion. The left ventricles were used for histological and biochemical studies.

### Experimental groups

The following experimental groups were studied:

1. LAD was occluded for 60 min. and re-perfused for 180 min. plus administration of 3,4-dihydro-5-[4-(1-piperidinyl) butoxy]-1(2H)-isoquinoli-none [DPQ_inhibitor of PARP) (Sigma, USA) [10 mg/kg i.p. 2 hrs before reperfusion. DPQ was dissolved in 100 μL dimethylsulfoxide (DMSO)] (*n***=** 24).
2. LAD was occluded for 60 min. and re-perfused for 180 min. plus administration of DMSO (100 μL DMSO were injected i.p. 2 hrs before reperfusion) (*n***=** 24).
3. LAD was occluded for 60 min. and reperfused for 180 min. plus administration of SP600125 (inhibitor of JNK) (Sigma, USA) (6 mg/kg i.p. 2 hrs before reperfusion. JNK was dissolved in 100 μL DMSO) (*n***=** 8).
4. LAD was occluded for 60 min. and reperfused for 180 min. plus administration of DMSO (100 μL DMSO were injected i.p. 2 hrs before reperfusion.) (*n***=** 8).
5. Sham operation. (*n***=** 32).

### Determination of myocardial infarct size

At the end of reperfusion, the coronary artery was re-occluded and the heart was perfused with 3 ml of Evans blue to delineate myocardial area at risk (AAR). Then the hearts were harvested and rinsed in normal saline. The atria, right ventricle and great vessels were removed. Then the tissue was semi-freezed for 30 min. in a −20°C freezer in order to be cut more easily. After that, the left ventricle was surgically isolated and cut into slices (1 mm thick). Nitro blue tetrazolium was obtained in powder form (Sigma, USA). The tetrazolium powder was diluted in a phosphate buffer. We used a two part buffer system consisting of NaH2PO4 (0.1 M) and Na2HPO4 (0.1 M). The ratio of the volume of NaH2PO4 and Na2HPO4 in the buffer was 77.4% and 22.6%. The pH of the buffer was 7.4. The slices were then incubated in the solution at a temperature of 37°C for 5 min. After evaluating the entire surface area, segments with blue staining were designated as viable, and those without staining were designated as non-viable (infarcted). Finally the different areas of the ventricle were weighed separately. Myocardial ischaemic area (AAR) was expressed as the percentage of the left ventricle. Infarct size was expressed as the percentage of the AAR [20].

### Measurement of cardiac function

To monitor cardiovascular function during the I/R protocol, a fluid-filled cannula was introduced *via* the carotid artery into the left ventricle. LVDP, maximum rate of left ventricular pressure development (+dP/*dt*) and maximum rate of left ventricular pressure decline (-dP/*dt*) was measured using a pressure transducer interfaced with a computerized heart performance analysis system (Digi-Med, USA).

### Terminal deoxynucleotidyltransferase-mediated dUTP nick end labelling (TUNEL)

To evaluate apoptotic activity, the TUNEL technique was used. Each section was de-paraffinized and re-hydrated with serial changes of xylene and ethanol. Proteinase K (20 mg/l) was applied to the section for 30 min. with the intention of producing optimal proteolysis. The endogenous peroxidase was inhibited with 3% hydrogen peroxide for 10 min. A commercial apop-tosis detection kit (Roche, Germany) was used. The TdT reaction was carried out for 1 hr at 37°C, and then 3, 3′-diaminobenzidine (DAB) chromogen was applied. Haematoxylin- Eosin was used as a counter-stain. TUNEL-positive cells were determined by randomly counting ten fields of the section and were expressed as a percentage of normal nuclei [21].

### Preparation of the mitochondrial and nuclear fractions

Heart mitochondria and nuclear fractions were used to evaluate the activation of AIF by western blot analysis, respectively. They were prepared by differential centrifugation, which was described by Kim GT *et al.*[12]. Briefly, the ischaemic myocardium of killed rats were removed and cut into small pieces. The samples were suspended in six volumes of buffer (210 mM mannitol, 70 mM sucrose, 10 mM Tris-HCl, pH 7.5, 10 mM KCl, 1.5 mM MgCl2, 5 mM EDTA, 0.5 mM dithiothreitol, and 1 mM Na3VO4). After homogenized, the homogenates were centrifuged at 800 *g* at 4°C for 10 min. The pellets were nuclear fractions. The supernatant was centrifuged at 1,200 gat 4°C for 10 min. After decanting, it was re-centrifuged at 15,000 gat 4°C for 20 min., the pellets were mitochondrial fractions.

### Western blot

Myocardial PAR, JNK and AIF in the ischaemic myocardium after 180 min. reperfusion were determined by western blot analysis. Equal amounts of protein (50 **|x**g) were fractionated on 10% (for PAR) or 14% (for JNK and AIF) SDS-polyacrylamide gels in running buffer [25 mM Tris, 192 mM glycine, 0.1% (wt/vol) SDS, pH 8.3] at 120 V and then electroblotted to nitrocellulose membranes. Membranes were blocked at room temperature with 5% non-fat milk in Tris-buffered saline (25 mM Tris, 137 mM NaCl, and 2.7 mM KCl) containing 0.05% Tween-20 and then incubated overnight at 4°C with the following primary antibodies: rabbit polyclonal anti-rat AIF antibody (Cell Signaling Technology, USA. Dilutions: 1:1000. Weight molecular of AIF: 57 kD), rabbit polyclonal anti-rat phospho-SAPK/JNK (Thr183/Tyr185) antibody (Cell Signaling Technology, USA. Dilutions: 1:500. Weight molecular of p-JNK: 54 kD and 46 kD), rabbit polyclonal anti-rat SAPK/JNK antibody (Cell Signaling Technology, USA. Dilutions: 1:1000, Weight molecular of JNK: 54 kD and 46 kD), rabbit polyclonal anti-rat PAR antibody (Alexis Biochemichals, San Diego, CA, USA. Dilutions: 1:1000) and β-actin (Santa Cruz, USA. Dilutions: 1:2000, Weight molecular of β-actin: 42 kD). Then the membranes were washed three times in Tris-buffered saline Tween (TBST) and incubated with horseradish peroxidase-conjugated goat anti-rabbit secondary antibody (Santa Cruz, USA. Dilutions: 1:2000) at room temperature. Immunoreactive bands were visualized using an enhanced chemoluminescence and quantified by image analyser (AlphaImager 2200, USA).

### Statistics

Values were expressed as means **±** SD. Statistical analysis of the results was carried out by one-way analysis of the variance followed by the Newman-Keuls test. *P*<0.05 was considered significant.

## Result

### PARP, JNK and AIF were activated in the rat model of myocardial ischaemia and re-perfusion

**Fig. 1:**
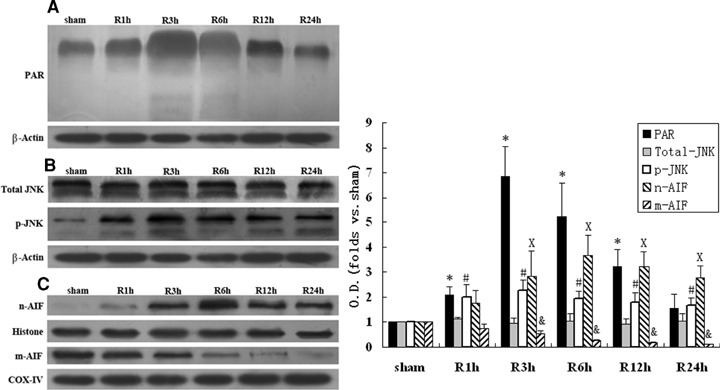
Western blot analysis of PAR (**A**), JNK (**B**) and AIF (**C**) in rats' hearts subjected to I/R. PAR and phospho-JNK increased during the I/R protocol. The activation of AIF increased in nuclear fractions while it decreased in mitochondria fractions. The peak activation of PARP was at 180 min. of reperfusion (*P<0.05 *versus sham*, #P<0.05 *versus sham*, ^X^P<0.05 *versus sham*, ^&^P<0.05 *versus sham*); (n-AIF: AIF of nucleus; m-AIF: AIF of mitochondria).

Western blot analysis was performed to evaluate whether PARP, JNK and AIF can be activated in the myocardial I/R rat model. Six rats hearts were studied in this group. As shown in Fig. 1, PAR and JNK increased during the I/R protocol. The activation of AIF also increased in nuclear fractions while it decreased in mitochondria fractions. The results of our experiment indicate that AIF transposed to the nucleus in the myocardial I/R rat model. From the fluctuations of PAR, JNK and AIF during the I/R protocol, it showed clearly the peak activation of PARP was at 180 min. of reperfusion. More interestingly, AIF also activated at 180 min. of reperfusion in nuclear fractions. Therefore, a rat model of LAD occlusion 60 min. and reperfusion 180 min. was selected in the successive study.

### Inhibition of PARP activity reduced myocardial infarct size

**Fig. 2:**
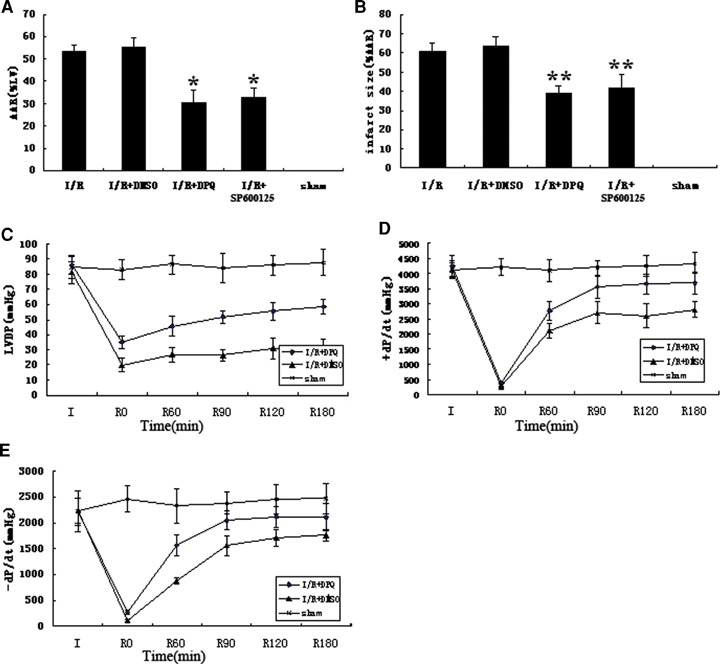
Changes of myocardial area at risk (**A**), infarct size; (**B**), LVDP; (**C**), LV ^+^dP/dt; (**D**), and LV -dP/dt (**E**), of different groups (sham, I/R, I/R+DMSO, I/R+SP600125 and I/R+DPQ) in rats' hearts. The myocardial area at risk and infarct size was similar between I/R and I/R+DMSO groups. A significant reduction in myocardial area at risk and infarct size was observed after PARP or JNK were inhibited (*P<0.05 *versus* I/R group, **P<0.05 versus I/R group). The sham group maintained the same LVDP and ±dP/dt throughout the protocol. The LVDP and ±dP/dt of I/R+DMSO group exhibited a significant reduction compared with sham group. The LVDP and ±dP/dt of I/R+DPQ group were significantly higher than I/R+DMSO group (*P*<0.05) but lower than sham group (*P*<0.05).

As shown in Fig. 2A and B, the AAR and infarct size of the heart were 52.95±1.28% and 61.11±7.46%, respectively in I/R group (*n***=** 6). These values showed no significant differences between I/R and I/R+ DMSO groups (*n***=** 6). Administration of DPQ or SP600125 caused significant reduction of AAR and infarct size. The AAR and infarct size of the heart was 31.12±1.58% and 38.83 + 5.67%, respectively in I/R+DPQ group (*n***=** 7). In I/R+SP600125 group (*n***=** 6), the AAR and infarct size of the heart was 32.61**±**4.16% and 41.52± 4.20%, respectively. These data suggest that inhibition of PARP or JNK activity reduces myocardial infarct size in rat heart I/R injury.

### Inhibition of PARP activity protected cardiac function

Figures 2C-E contains the mean values of LVDP, +dP/dt and -dP/dt during preischaemia, ischaemia and re-perfusion in animals from all experimental groups. No group differences existed in any of these values before the introduction of ischaemia. However, during ischaemia and re-perfusion, the LVDP values of I/R+ DMSO group (*n***=** 6) were significantly lower than those of the sham group (*n***=** 8) (P<0.05). The LV ±dP/dt values of I/R+DMSO group were also significantly lower than those of the sham group (P<0.05). Both LVDP and LV ± dP/dt values of I/R+DPQ group (*n***=** 7) were significantly higher than I/R+DMSO group. These preliminary data indicate that inhibition of PARP activity may play an essential role in the protection of cardiac function in rat heart I/R injury.

### Inhibition of PARP reduced the apoptosis of cells of the heart

**Fig. 3:**
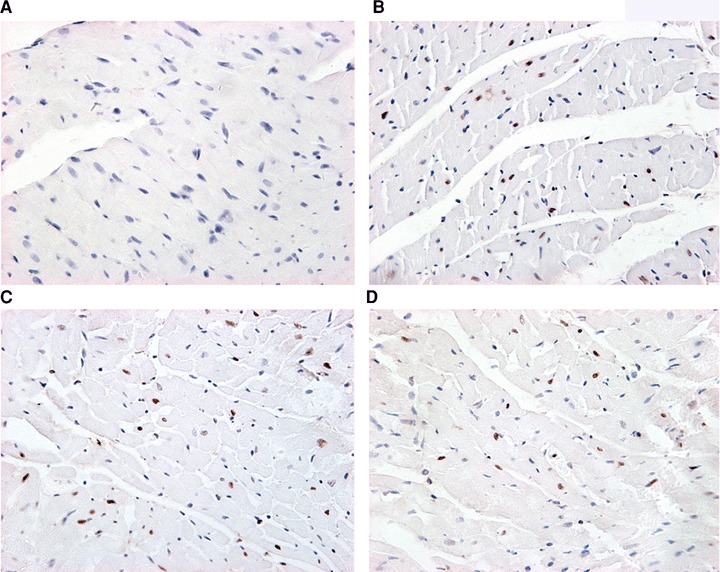
Representative photomicrographs of ventricular tissue stained for TUNEL for DNA breaks. (**A**): Sham group; (**B**): I/R group; (**C**): I/R+DMSO group; (**D**): I/R+DPQ group. In I/R and I/R+DMSO groups a large number of TUNEL positive cells are observed subsequent to ischaemia and reperfusion injury. The TUNEL positive cells of I/R+DPQ group were significantly reduced compared with I/R group or I/R+DMSO group (magnification, 400x).

Figure 3 showed the TUNEL positive cells. TUNEL positive cells were expressed as a percentage of normal nuclei. No TUNEL positive cells were found in the sham group (*n***=** 8). However, the number of TUNEL positive cells was significantly increased in I/R group (35±5.3%) (*n***=** 7) and I/R+DMSO group (37±2.1%) (*n***=** 7). The TUNEL positive cells were significantly reduced to 20±4.1% in the I/R+DPQ group (*n***=** 8) (P<0.05 versus I/R group). These data suggest that inhibition of PARP activity reduces cells apoptosis in rat heart I/R injury.

### Inhibition of PARP activity reduced the activation of JNK

**Fig. 4:**
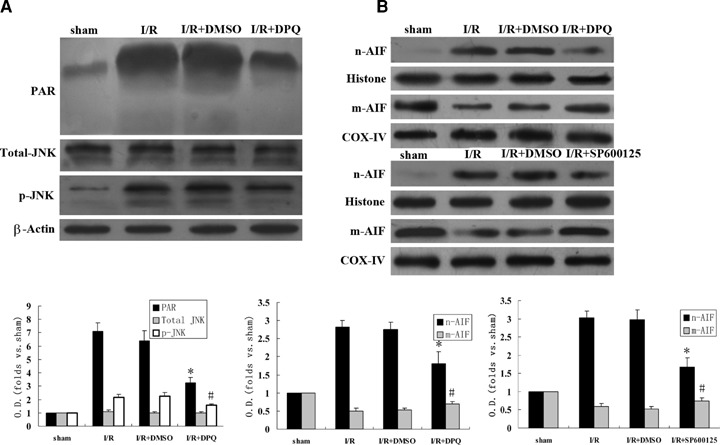
Representative western blot analysis of PAR, JNK and AIF of different groups (sham, I/R, I/R **+** DMSO, I/R **+** DPQ and I/R+SP600125). (**A**) The activation of PAR and phospho-JNK increased in I/R and I/R+DMSO groups. In I/R+DPQ group, the activation of PAR and phospho-JNK was significantly attenuated compared with I/R group or I/R+DMSO group (*P<0.05, ^#^*P*<0.05). (**B**) The activation of AIF increased in I/R and I/R+DMSO groups in heart nuclear fractions. After administration of DPQ or SP600125, the activation of AIF was significantly attenuated in heart nuclear fractions (*P<0.05 versus I/R group, ^#^P<0.05 versus I/R group). In heart mitochondria fractions, the activation of AIF increased after administration of DPQ or SP600125 (*P<0.05 versus I/R group, ^#^*P*<0.05 versus I/R group).

Western blot analysis was performed to examine whether the activation of JNK in the rat model of myocardial I/R was related with PARP. As shown in Fig. 4A, phospho-JNK increased after reperfusion in non-inhibition of PARP rat. Ischaemia and reperfusion-induced activation of phospho-JNK was significantly attenuated in the inhibition of PARP. These data suggest that JNK may be downstream of PARP activation in rat heart I/R injury.

### Inhibition of PARP or JNK activity reduces the AIF translocation

As shown in Fig. 4B, AIF increased after reperfusion in nuclear fractions. The activation of AIF was significantly attenuated by inhibition of PARP or JNK activity. On the contrary, AIF decreased in mitochondria fractions after reperfusion. Inhibition of PARP or JNK activity could increase the activation of AIF in mitochondria fractions. These data suggest that inhibition of PARP or JNK activity was able to reduce the mitochondrial-nuclear translocation of AIF. These preliminary data indicate AIF may be downstream of PARP and JNK activation. JNK may play an essential role in PARP-mediated AIF translocation.

## Discussion

In this experiment, we used DPQ to inhibit the activation of PARP in the rat model of myocardial ischaemia and reperfusion *in vivo*; as a result, the infarct size and the apoptosis of cells of hearts reduced, the cardiac function was protected. At the same time, the activation of JNK and the translocation of AIF from mitochondrial to nuclear were attenuated. We also found that inhibition of the activation of JNK was able to attenuate the translocation of AIF from mitochondrial to nuclear. The present study demonstrated that inhibition of PARP can reduce heart I/R injury *in vivo*. In this pathophysiological course, PARP may regulate the translocation of AIF by the activation of JNK.

Coronary reperfusion after ischaemia results in a marked cellular injury and a large infarct rea, which ultimately leads to heart failure. Pathophysiological states, such as inflammation [22], circulatory shock [23] and I/R injury generate free radicals and oxidant species leads to DNA injury and the activation of PARP. In this experiment, western blot analysis showed that PARP increased during the reperfusion (up to 24 hrs) protocol. The results conform to those of other researches' [24]. Our experiment also showed that the peak activation of PARP was at 180 min. of re-perfusion. Several independent studies have confirmed that pharmacological inhibition of PARP [24–26] significantly reduced the infarct size and improved the cardiac function in I/R injury. In this experiment, we used DPQ to inhibit the activation of PARP. As a result, the infarct size of hearts reduced and the cardiac function was protected. DPQ is a very potent PARP inhibitor [27]. Xu *et al.*[19] in their experiment also used DPQ to inhibit PARP. The cardiac protection afforded by the PARP inhibitors was due to a selective inhibition of PARP Though PARP was proposed to play a role in the pathogen-esis of heart I/R injury, the precise mechanisms of PARP activation in heart I/R injury were not fully understood. Studies proved that mechanisms of PARP activation in heart I/R injury may involve several aspects. The fundamental pathologic effect of PARP activation is related to the myocardial energy metabolism [3, 4]. In heart I/R injury the hyper-activation of PARP is able to disturb the energy generation process and the mitochondrial function. PARP can also regulate the expression of various proteins and cytokines such as ICAM-1 [28], AP-1 complex [29], nf-kb[30], TNF-α and IL-10 [31]. PARP inhibition or PARP deficiency have been shown to suppress the cytokines expression *in vitro* or *in vivo*.

AIF is a 67 kD death promoting protein [32]. In healthy cells it is normally confined to mitochondria. Upon death stimuli, AIF transposes from the mitochondria to the nuclei. As a result, large-scale DNA break, and cells die. However, van Empel *et al*. [33] reported that AIF exerts also cytoprotective effects on myocytes both *in vitro* and *in vivo*. They found that AIF has a role of antiox-idant in stress–induced cell death and development of heart failure induced by pressure overload. But the precise mechanism for AIF protection in these cells was not known. van Empel presumed that AIF plays a critical role in mitochondrial respiration, by handling of reactive oxygen radicals that are normally released by the respiratory chain. In our study, we found that AIF had a proapoptotic role in rat heart I/R injury.

Recent studies demonstrated that AIF transposed from the mitochondria to the nuclei required PARP activity [14, 16]. These studies demonstrated that AIF was a downstream factor in PARP-mediated cell death. In this experiment, AIF increased after reperfusion in nuclear fractions. The activation of AIF was significantly attenuated by inhibition of PARP activity. These preliminary data indicate AIF may be downstream of PARP activation. However, how PARP regulates or induces AIF translocation or release from the mitochondria is not known. XU *et al.*[19] in their experiment found, based on genetic knockouts and pharmacological inhibition, that JNK, but not the other groups of mitogen activated protein kinase (MAPK), is required for PARP-induced mitochondrial dysfunction, AIF translocation and subsequent cell death *in vitro*. JNK has recently been demonstrated to be involved in cell apoptosis and necrosis [34]. JNK activation is also an important signaling step leading to cardiac myocytes apoptosis [35]. *In vivo* studies demonstrated that myocardial ischaemia followed by reperfusion in the heart were able to cause activation of JNK [36, 37]. Our data of the present study also showed that JNK activated in I/R hearts and JNK may participate in the PARP-mediated release of AIF.

In this experiment, we found that the inhibition of PARP was able to reduce the heart infarct size and the apoptosis of cells in heart I/R injury *in vivo*. We further elucidated that the translocation of AIF from mitochondrial to nuclear attenuated after blocking PARP or JNK. Therefore, our results indicate that JNK may be downstream of PARP activation and JNK may be required for PARP mediates AIF translocation in a rat model of myocardial ischaemia and reperfu-sion *in vivo*. PARP/JNK/AIF pathway may be a new pathway of cardiac myocyte injury *in vivo*. This may provide a new management for patients suffering coronary heart diseases in the future.
